# Time Course Analysis of Skeletal Muscle Pathology of GDE5 Transgenic Mouse

**DOI:** 10.1371/journal.pone.0163299

**Published:** 2016-09-22

**Authors:** Takao Hashimoto, Bo Yang, Yuri Okazaki, Ikumi Yoshizawa, Kaori Kajihara, Norihisa Kato, Masanobu Wada, Noriyuki Yanaka

**Affiliations:** 1 Graduate School of Biosphere Science, Hiroshima University, Higashi-Hiroshima, Japan; 2 Graduate School of Integrated Arts and Sciences, Hiroshima University, Higashi-Hiroshima, Japan; University of Minnesota Medical Center, UNITED STATES

## Abstract

Glycerophosphodiesterase 5 (GDE5) selectively hydrolyses glycerophosphocholine to choline and is highly expressed in type II fiber-rich skeletal muscles. We have previously generated that a truncated mutant of GDE5 (GDE5dC471) that lacks phosphodiesterase activity and shown that transgenic mice overexpressing GDE5dC471 in skeletal muscles show less skeletal muscle mass than control mice. However, the molecular mechanism and pathophysiological features underlying decreased skeletal muscle mass in GDE5dC471 mice remain unclear. In this study, we characterized the skeletal muscle disorder throughout development and investigated the primary cause of muscle atrophy. While type I fiber-rich soleus muscle mass was not altered in GDE5dC471 mice, type II fiber-rich muscle mass was reduced in 8-week-old GDE5dC471 mice. Type II fiber-rich muscle mass continued to decrease irreversibly in 1-year-old transgenic mice with an increase in apoptotic cell. Adipose tissue weight and blood triglyceride levels in 8-week-old and 1-year-old transgenic mice were higher than those in control mice. This study also demonstrated compensatory mRNA expression of neuromuscular junction (NMJ) components, including nicotinic acetylcholine receptors (α1, γ, and ε subunits) and acetylcholinesterase in type II fiber-rich quadriceps muscles in GDE5dC471 mice. However, we did not observe morphological changes in NMJs associated with skeletal muscle atrophy in GDE5dC471 mice. We also found that HSP70 protein levels are significantly increased in the skeletal muscles of 2-week-old GDE5dC471 mice and in mouse myoblastic C2C12 cells overexpressing GDE5dC471. These findings suggest that GDE5dC471 mouse is a novel model of early-onset irreversible type II fiber-rich myopathy associated with cellular stress.

## Introduction

The physiological process of aging critically affects an individual's quality of life via gradual functional, structural, and biochemical changes. Age-related decline in skeletal muscle mass and functions is defined as sarcopenia, a condition characterized by muscle weakness and fiber atrophy [1–3]. Persons with sarcopenia suffer greater incidence of functional disabilities that lead to loss of independence and are more likely to suffer injuries due to falling [4]. Thus, sarcopenia is a strong predictor of mortality in aging individuals. Few rodent models exhibit age-related changes in skeletal muscles spontaneously and, therefore, long-term examination is required to characterize muscle weakness and fiber atrophy during aging and senescence in these systems. For example, age-related skeletal muscle pathology develops in about 28 months in F344/Brown-Norway F1 hybrid rat [5], and a senescence-accelerated SAMP1, SAMP6 or SAMP8 mouse [6–8]. An animal model with sarcopenia-like muscle atrophy that develops more quickly is urgently needed to advance studies of this degenerative condition.

Most skeletal muscles consist of intermixed mitochondria-rich slow-twitch fiber (type I) and fast-twitch muscle fiber (type II) [9]. It was reported that aging skeletal muscles were more atrophied in type II muscle fiber than in type I muscle fiber [9–12]. These muscle fibers are controlled by different motor units during young adulthood and are repeatedly denervated and reinnervated through adulthood to old age, and in very old age, skeletal muscle atrophy may be due to increasingly frequent axonal degeneration and/or motor cell death [13], (See review in [14]). The relationship between skeletal muscle atrophy and decreased neuromuscular transmission has been recently discussed in rodent models and humans [15, 16]. Previous studies showed that neuromuscular junctions (NMJs) in skeletal muscles of aged rats are denatured before the onset of muscle atrophy and that NMJs of type II fibers are more prone to denaturation than those of type I fibers [15, 17]. On the other hands, mRNA expression of nicotinic acetylcholine receptor (nAchR) subunits, which compose a pentameric nAchR and are localized to NMJs, increases with age [18–20]. Similarly, expression of the nAchR subunit was transiently up-regulated during an acute inactivity period in rodent models with sciatic denervation or spinal cord injuries [22–24]. These observations suggested that the expression of components localized at the NMJs have compensatory effects on skeletal muscle dysfunction. Although type II-rich fiber atrophy has been reported in hindlimb suspension, plaster cast immobilization, or short-term spaceflight induced muscle disuse rodents which are independent of denervation [25, 26], the alternation of NMJ functions during type II-rich skeletal muscle atrophy is little understood. Some mechanisms such as oxidative stress and chronic inflammation have been proposed for skeletal muscle atrophy [27–29], whereas the pathophysiological relationship between NMJ functions and decreased physical activity during type II-rich skeletal muscle atrophy also remains unclear.

We recently generated transgenic mice with selectively inducible skeletal muscle atrophy in type II-rich gastrocnemius and quadriceps muscles at around 12 weeks [30]. These mice overexpressed a truncated mutant of glycerophosphodiesterase 5 (GDE5) that lacks phosphodiesterase activity. mRNA expression analyses of the quadriceps of 12-week-old GDE5dC471 mice indicated that mRNAs for inflammatory cytokines and cellular defense were up-regulated [30] and that the skeletal muscle atrophy is independent of the ubiquitin-proteasome system [30, 31]. Interestingly, the expression of several genes linked to NMJs was up-regulated in the skeletal muscle of the GDE5ΔC471 mice, thus resembling age-related skeletal muscle atrophy. However, the molecular mechanisms underlying the decreased skeletal muscle mass and pathophysiological characteristics as a new animal model for skeletal muscle atrophy remain unclear.

The aim of this study was to reveal the pathology and underlying cause of type II-rich skeletal muscle atrophy in GDE5dC471 mice skeletal muscles by examining cross-sections of myofibrils, muscle functions, and neuromuscular junctions through time. This study provides detailed information on the skeletal muscle pathology of GDE5dC471 mice as a useful animal model for drug development and evaluation of functional food products that protect against the onset/progression of type II-rich skeletal muscle atrophy.

## Materials and Methods

### Animals

GDE5dC471 transgenic mouse was generated as described previously [30]. In this experiment, heterozygous male mice harboring the transgene which were backcrossed at least 5 times with purebred C57BL/6J females (Charles River Japan, Kanagawa, Japan), and their male control litter mates were used. The presence of GDE5dC471 transgenes in offspring was determined by polymerase chain reaction (PCR) with the following primers: GDE5dC471, sense, 5'-TTTGATGTCCACTTTCAAAGGAC-3', and antisense, 5'-CTCCATCCCTGTGTTGGCAAATCC-3'. Mice were housed in a room maintained at 24°C with a 12-hr light and dark cycle (lights on, 8:00 a.m. to 8:00 p.m.), and MF solid chow (Oriental Yeast, Tokyo, Japan) and a bottle tapped deionized water were provided ad libitum. The animal study was approved by the Hiroshima University Animal Committee (Permit Number: C15-9-2), and the mice were maintained in accordance with the Hiroshima University Guidelines for the Care and Use of Laboratory Animals. After 2-, 4-, 8-weeks or 1-year rearing periods, blood samples were collected from mice by venipuncture from the aorta under sodium pentobarbital anesthesia and were sacrificed, followed by removal of skeletal muscles (gastrocnemius, quadriceps or soleus) or epididymal and perirenal white adipose tissues.

### Histopathology

To measure cross-sectional muscle fiber area, samples of the skeletal muscle (gastrocnemius) of GDE5dC471 mice and age-matched control mice at 4 and 8-week, and 1-year old of age were fixed in neutral buffered 10% formalin, and processed by staining paraffin-embedded transverse sections (2 μm) with hematoxylin and eosin (H&E). To detect terminal deoxynucleotidyl transferase (TdT) dUTP nick-end labeling (TUNEL) positive nuclear in gastrocnemius muscle of 4-week-old (frozen section), 8-week-old and 1-year-old (paraffin section) GDE5dC471 mice and age-matched control mice were subjected. Frozen section for TUNEL staining was prepared that the gastrocnemius muscle was harvested, fixed in 4% paraformaldehyde for 1 day, then transferred to Holt's hypertonic gum-sucrose medium (0.88M sucrose containing 1% gum acacia) for 2Days at 4°C, embedded in optimum cutting temperature compound and immediately frozen in liquid nitrogen-cooled isopentane (Sigma-Adrich Japan, Tokyo, Japan), then prepared as 6 μm sections using a cryostat. TUNEL staining was performed using ApopTag® Peroxidase In Situ Apoptosis Detection Kit (Merck Millipore, Temecula, CA) according to the manufacturer's instructions. 3, 3'-diaminobenzidine (DAB) was used as peroxidase substrate, and for calculate the ratio of TUNEL positive nuclear to total myonuclei (%), counterstaining with hematoxylin was also performed. In order to observe morphology of nicotinic acetylcholine receptor, gastrocnemius muscles of 1-year-old GDE5dC471 mice and control mice were frozen in liquid nitrogen-cooled isopentane (Sigma-Adrich Japan, Tokyo, Japan), and longitudinal sections (20 μm) embedded in optimum cutting temperature compound were stained with fluorescent α-bungarotoxin Alexa Fluor®594 (Molecular Probes, Eugene, OR)

### PCR analyses

Semiquantitative and quantitative PCR analyses were performed on total RNA, which was extracted from dissected quadriceps muscles of 2-, 4-, 8-week and 1-year-old GDE5dC471 and age-matched control mice, prepared with RNeasy Lipid Tissue Mini Kit (Qiagen, Hilden, Germany) according to the instructions of the manufacturer. The reverse transcriptase reaction was carried out with 1 μg of total RNA as a template to synthesize cDNA using RevaTra Ace (Toyobo, Osaka, Japan) reverse transcriptase. For semiquantitative PCR analysis, cDNA and specific primers were added to the GoTaq Master Mix (Promega, Madison, WI) to give a total reaction volume of 20 μl. The reactions were sampled after 25, 28, and 30 cycles under different PCR conditions to monitor product accumulation. For quantitative PCR analysis, cDNA and specific primers were added to THUNDERBIRD™SYBR ®qPCR Mix (Toyobo) to give a total reaction volume of 15 μL. PCR reactions were performed using StepOnePlus^TM^ (Applied Biosystems, Foster City, CA). The PCR reactions were initiated with denaturation for 10 min at 95°C, followed by amplification with 40 cycles each for 15 s at 95°C, and extended for 1 min at 60°C. The primers used for semiquantitative RT-PCR or realtime PCR analysis were as follows: Acsm3, sense, 5’-CCTGATCCTATGAGAGTCTTG-3’, and antisense, 5’-GCAGTATCACCATTACTCTGTC-3’; Slc27a2, sense, 5’-GCGGCAACCATCAATCATCA-3’, and antisense, 5’-CGGTGTGTTGCACAGGTACC-3’; nAchRα1, sense, 5′-GCCGGACGTCGTTCTCTATA-3′, and antisense, 5′-GTAGAACACCCAGTGCTTCC-3′; nAchRε, sense,5′-GCGTGCTCATTTCTGGCTTG-3′, and antisense, 5′-CGCGGCAGCAGCTCTAATAA-3′; nAchRγ, sense, 5′-CAAAGGCAGCGCAATGGATT-3′, and antisense, 5′-GTAGTGGGCCATGAGGAAGA-3′; Ache, sense, 5′-GCTCCTACTTTCTGGTTTAC-3′, and antisense, 5′-AAAGATGTAGGCATAGACCC-3′; L19, sense, 5′-GGCATAGGGAAGAGGAAGG-3′, and antisense, 5′-GGATGTGCTCCATGAGGATGC-3′; β-actin, sense, 5’-TTGGGTATGGAATCCTGTGGCATC-3’, and antisense, 5’-CGGACTCATCGTACTCCTGCTTGC-3’. Realtime PCR samples were normalized according to L19 mRNA levels.

### Blood Analysis

Six hours before blood collection, food was withdrawn from mice. Serum was obtained by centrifugation of the whole blood sample at 900 × g for 10 min at 4°C and stored at -20°C for late use. Serum glucose, triglycerides and total-cholesterol were measured by Hitachi 7180 Biochemistry Automatic Analyzer (Hitachi, Tokyo, Japan).

### Rotarod test

Motor coordination in 8-week and 1-year-old GDE5dC471 and control mice was assessed by KN-75 rotating rod apparatus (Natsume Seisakujo, Tokyo, Japan), which consisted of a plastic rod (3 cm diameter, 8 cm length) with a gritted surface flanked by 2 large discs. Each mouse was placed on the rod rotated 15 times per minute, and latency to fall from the rod was recorded.

### DNA Microarray

DNA microarray analysis was performed in accordance with our previous study [30]. Total RNA derived from the skeletal muscle (quadriceps) of GDE5dC471 and control mice at 2 weeks of age was isolated using RNeasy Lipid Tissue Mini Kit and subjected to cRNA synthesis for a DNA microarray analysis according to the manufacturer’s instructions (whole mouse genome 60-mer oligo microarray, Agilent Technologies, Santa Clara, CA). All of the procedures of fluorescence labeling, hybridization, and slide and image processing were carried out according to the manufacturer’s instructions. Briefly, aliquots of cRNA samples were fragmented and hybridized on the whole mouse genome oligo microarray slides at 65°C for 17 h. The slides were then sequentially washed, dried, and scanned using an Agilent DNA microarray scanner with Sure Scan technology (Agilent Technologies). In this study, the DyeSwap method was used to eliminate the bias between dyes. Gene expression data were obtained with Agilent Feature Extraction software, using defaults for all parameters except ratio terms, which were changed according to the Agilent protocol to fit the direct labeling procedure. Files and images, including error values and p values, were exported from Agilent Feature Extraction software (version 9.5). The microarray data are also deposited in the NCBI GEO data base (available on the World Wide Web at www.ncbi.nlm.nih.gov/geo) under accession number GSE80704.

### Western blot analysis

Gastrocnemius samples were homogenized in TNE buffer (20mM Tris-HCl, pH 7.5, 0.15 M NaCl, 1 mM EDTA and 0.5% NP-40) using a Polytron homogenizer. The homogenates were centrifuged at 12000 rpm for 15 min at 4°C. Protein concentrations of the supernatants were determined using the BioRad Detergent Compatible (DC) assay kit (BioRad). Tissue lysates containing 16 μg protein were separated SDS–PAGE using a 10% polyacrylamide gel and stained with Coomassie Brilliant Blue (CBB) for determination of separated protein concentration or transferred to Immobilon P filters (Millipore, Bedford, MA). The filters were blocked for 2 h at room temprature by soaking in 4% nonfat dried milk (Nacalai Tesque, Kyoto, Japan) in PBS; then they were incubated for 18 h at 4°C with the anti-HSP70 mouse monoclonal antibody (Santa Cruz Biotechnology, Santa Cruz, CA) and after incubation in peroxidase labeled anti-mouse secondary antibody (Sigma-Adrich Japan) for 1 h the proteins were detected using ECL Western Blotting Detection Reagents (GE healthcare Japan, Tokyo, Japan). The signals were visualized by exposing the membranes to X-ray films (Fujifilm, Tokyo, Japan).

### Statistical analysis

To determine significant differences in pathophysiology, parametric comparisons between GDE5dC471 mice and age-matched control mice were carried out the two-tail unpaired t-test. Time-course data was analyzed with a two-way ANOVA followed by the two-tail unpaired t-test for evaluation the statistical significance between GDE5dC471 mice and age-matched control mice at 4 and 8-week, and 1-year old of age. Statistical analysis was executed with Prism 7 version 7.01 software (GraphPad Software, Inc.). *p* values of < 0.05 (two sided) were considered statistically significant.

Supporting materials and methods were described in S1 File.

## Results

### Pathophysiological alterations of type II fiber-rich muscles in GDE5dC471 mice through time

**Fig 1:**
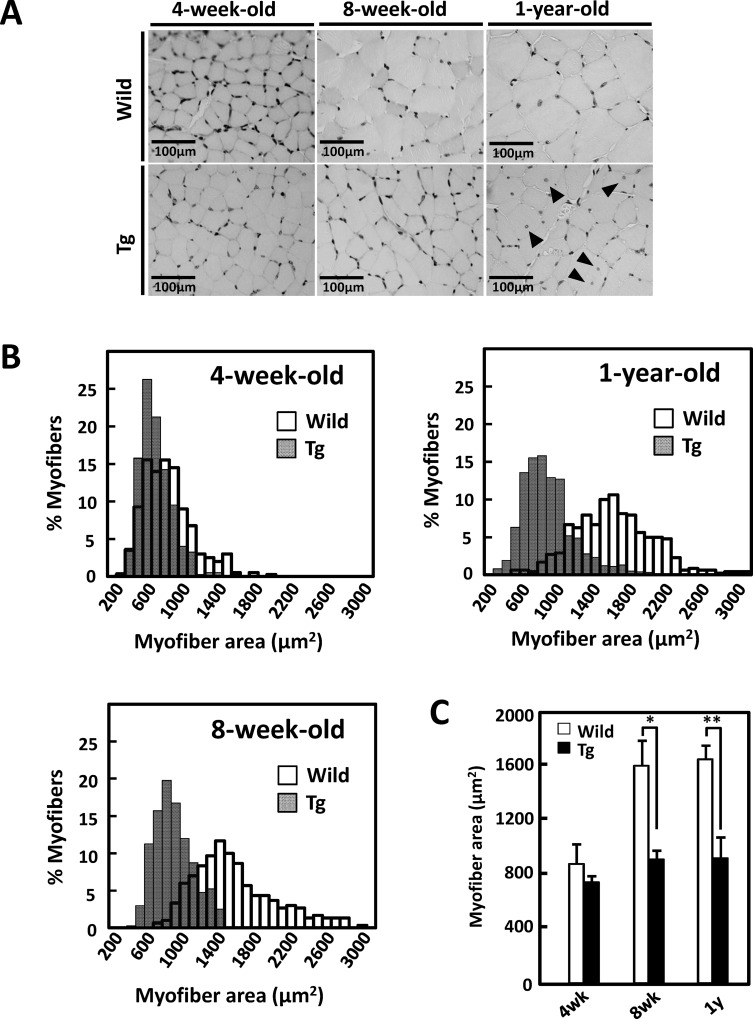
Time-course morphological alterations of type II fiber-rich muscles in GDE5dC471 mice. *A*, Representative photographs of H&E staining in cross sections of gastrocnemius muscle, Left; 4-week-old GDE5dC471 mice (Tg) and age-matched control mice (Wild), center; 8-week-old, and right; 1-year-old. Central nuclei (arrow) appeared only in 1-year-old GDE5dC471 mice. *B*, Time-course alterations of fiber areas of gastrocnemius muscle of 4-week-old GDE5dC471 mice (Tg) and age-matched control mice (Wild). To visualize frequency of distribution of each myofibril, histogram images were analyzed. Both 8-week-old and 1-year-old Tg showed a leftward shift, indicating an evident increase in the percentage of small areas compared with age-matched Wild. *C*, Mean fiber areas of gastrocnemius muscle of 4-week-old GDE5dC471 mice (Tg) and age-matched control mice (Wild). 4-week-old: n = 4, 8-week-old: n = 3, and 1-year-old: n = 7 (Wild) and n = 10 (Tg). Data represent mean ± SD. *p<0.05, **p<0.01.

**Fig 2:**
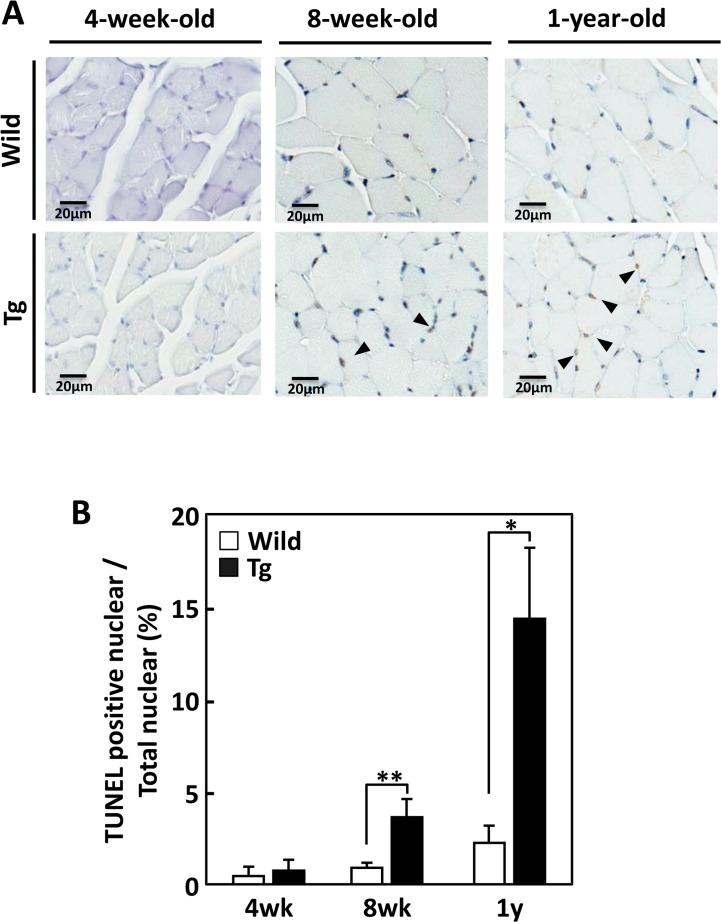
Time-course alterations of TUNEL staining positive nuclear in type II fiber-rich muscles of GDE5dC471 mice. A, Representative photographs of TUNEL staining in transverse sections of gastrocnemius muscle. Hematoxylin counter staining was performed after the TUNEL staining to clarify the TUNEL-positive nuclei under nuclear staining, Left; 4-week-old GDE5dC471 mice (Tg) and age-matched control mice (Wild), center; 8-week-old, and right; 1-year-old. Arrows indicated TUNEL-positive nuclear. B, Time-course increase in the ratio of TUNEL staining positive nuclear per total myonuclei in gastrocnemius muscles of GDE5dC471 mice and age-matched control mice. 4-week-old: n = 4, 8-week and 1-year-old: n = 5. Data represent mean ± SD. *p<0.05, **p<0.01.

We examined morphological alterations in type II fiber-rich muscle tissues of juvenile (4-week-old), young (8-week-old), and middle-aged (1-year-old) heterozygous GDE5dC471 mice and age-matched control mice. In 8-week-old and 1-year-old GDE5dC471 mice, gastrocnemius and quadriceps muscles were smaller than those of age-matched control mice (Table 1). Muscle weights did not differ significantly between 4-week-old GDE5dC471 mice and control mice. To quantify muscle shrinkage, we measured the cross-sectional areas of gastrocnemius muscles from GDE5dC471 and control mice (Fig 1A). Smaller fibers were more frequent in 8-week-old and 1-year-old GDE5dC471 mice (Fig 1A and 1B). Although the mean fiber cross-sectional area of 4-week-old GDE5dC471 mice was slightly smaller than in age-matched control mice, that of 8-week-old or 1-year-old GDE5dC471 mice was time-dependently reduced by 42.3% or 44.2% from control mice, respectively (Fig 1C). These results suggest that type II fiber-rich muscle atrophy had already developed in young (8-week-old), and was irreversibly observed through middle age. As noted also, centrally located myonuclei were evident in the type II fibers of 8-week-old and 1-year-old GDE5dC471 mice (Fig 1A and S1 Fig) and the number of TUNEL positive cells is time-dependently increased in the type II fibers of 8-week-old and 1-year-old GDE5dC471 mice (Fig 2). To examine the relationship between skeletal muscle atrophy and metabolic features, we measured white adipose tissue weight, and serum glucose, triglyceride and total cholesterol levels of GDE5dC471 mice (8-week-old and 1-year-old) and of age-matched control mice. Although there was no difference in serum glucose and total cholesterol levels between these mice, white adipose tissue weight and serum triglyceride levels in GDE5dC471 mice were higher than in control mice (Table 2). In order to investigate the skeletal muscle metabolism, we analyzed mRNA expression of α-glycerophosphate dehydrogenase (αGPD), which is an important enzyme for glycolysis, and of genes linked to mitochondria functions such as citrate synthase (Cs) and peroxisome proliferator-activated receptor (PPAR)-γ co-activator-1α (PGC1α) in skeletal muscles of GDE5dC471 mice. Although mRNA expression of mitochondrial markers, Cs and PGC1α, was not altered in muscles of GDE5dC471 mice, αGPD mRNA was significantly down-regulated in muscles of 1-year-old GDE5dC471 mice (S2A and S2B Fig). The decreased glucose metabolism may be involved in the metabolic complications such as increased adipose tissue weight and serum triglyceride level in GDE5dC471 mice. Furthermore, we analyzed type I (slow-twitch) and type II (fast-twitch) fiber mRNA expression in GDE5dC471 mice and could not observe fast- to slow-twitch fiber transformation in the gastrocnemius muscle of 1-year-old GDE5dC471 mice (S3 Fig).

**Table 1:** Time course alterations of body weight and skeletal muscle weight in GDE5dC471 mice and age-matched control mice.

Mice	Body weight (g)	Skeletal muscles (mg)		
		Quadriceps	Gastrocnemius	Soleus
4-week-old WT (n = 4, 3^$^)	15.3±0.4	77.7±4.2	77.8±5.5	3.5±0.4
4-week-old Tg (n = 4, 3^$^)	15.0±0.3	72.5±4.7	70.7±0.9	3.8±0.4
8-week-old WT (n = 3)	21.8±0.2	161.8±8.3	144.0±9.5	5.3±0.4
8-week-old Tg (n = 3)	21.7±0.5	101.3±3.0*	99.0±9.0*	4.7±0.6
1-year-old WT (n = 7)	33.3±0.9	205.0±9.6	181.0±3.6	8.7±1.1
1-year-old Tg (n = 10)	31.8±1.2	132.2±5.0**	122.5±4.4**	10.2±0.7

WT: control mice, Tg: GDE5dC471 mice

^$^: soleus muscle. Data represent means±SE

*: *p*<0.05

**: *p* <0.01 (unpaired t-test, versus age-matched WT)

**Table 2:** Obesity-related parameters in 8-week-old and 1-year-old of GDE5dC471 mice, and age-matched control mice.

Mice	WAT (mg)	GLU (mg/dL)	TG (mg/dL)	T-CHO (mg/dL)
8-week-old WT (n = 3)	258±80	269±10	30±2	72±4
8-week-old Tg (n = 3)	329±14.6*	276±7	57±7*	75±3
1-year-old WT (n = 5)	784±73	207±11	20±2	82±2
1-year-old Tg (n = 10)	1005±189	219±12	28±3*	82±5

WT: control mice, Tg: GDE5dC471 mice, WAT: White adipose tissue, GLU: serum glucose, TG: serum triglyceride, T-CHO: serum total cholesterol. Data represent means±SE

*: *p* <0.05 (unpaired t-test, versus age-matched WT)

**Fig 3:**
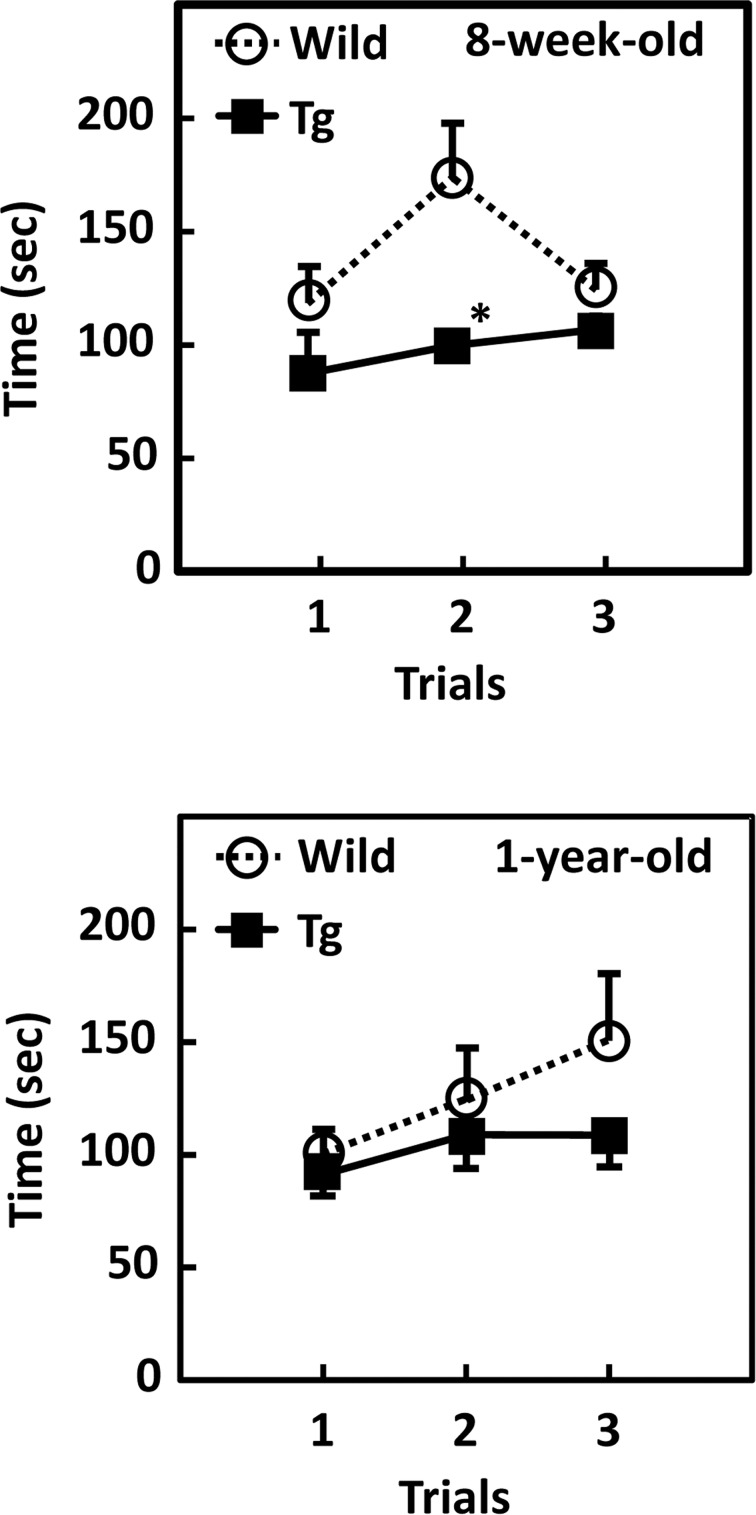
GDE5dC471 overexpression in skeletal muscle affects passive motion. Passive motion was examined by the rotarod performance test. Both 8-week-old and 1-year-old GDE5dC471 mice (Tg) revealed a shortening of duration at the rotating rod compared with age-matched control mice (WT) in all trials (8-week-old, n = 5; 1-year-old, n = 7). Data represent mean ± SE. * *p*<0.05.

To investigate whether GDE5dC471-related muscle atrophy affects passive motion, we performed a rotarod test using GDE5dC471 mice and age-matched control mice. GDE5dC471 mice demonstrated worse motor performance on the rotarod than control mice in all trials (Fig 3). Next, mice were transferred to cages with a running wheel and monitored for the number of wheel revolutions made for 3 days. The voluntary motor activity was not different between GDE5dC471 mice and control mice (S4 Fig). Because type I fibers are more responsible for the voluntary motor activity than type II fibers [32], these data were consistent with the result showing that soleus muscle weight was not different between GDE5dC471 and wild-type mice (Table 1). Taken together, these observations suggest that GDE5dC471 mice show not only morphological alteration of type II fiber muscles but also decreased motor coordination ability and that the reduction of type II fiber-rich muscle tissue mass may affect their physical performance.

### NMJ-related mRNA expression and nAchR morphology in GDE5dC471 mice

**Fig 4:**
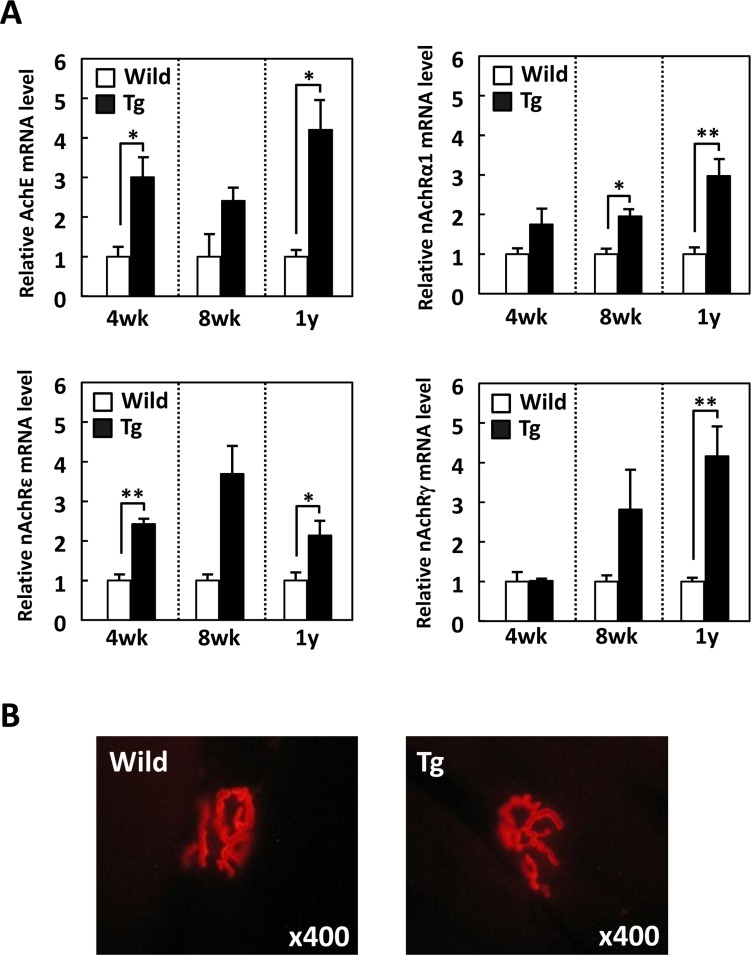
GDE5dC471 overexpression in skeletal muscle increases neuromuscular junctions-related mRNA expression. *A*, Total RNA from gastrocnemius muscle of GDE5dC471 mice (Tg) and age-matched control mice (Wild) (4-week-old, n = 4; 8-week-old, n = 3; and 1-year-old, n = 5) was subjected to quantitative PCR to examine mRNA expression level of genes related to neuromuscular junctions. Data represent mean ± SE. *p<0.05, **p<0.01. *B*, Representative photographs of fluorescent α-bungarotoxin staining in transverse sections of gastrocnemius muscle, Left; 1-year-old of GDE5dC471 mice (Tg), and right; age-matched control mice (Wild). No morphological alteration of nicotinic acetylcholine receptor (nAchR) was observed between GDE5dC471 and control mice.

In various murine models of skeletal muscle atrophy including age-associated myopathy, several genes related to NMJs were up-regulated in skeletal muscles [18–24, 33], suggesting that the compensatory expression of NMJ components contributes to muscle contraction and has preventive effects on skeletal muscle dysfunction. To evaluate whether the decreased skeletal muscle mass in GDE5dC471 mice affects the expression of NMJ components, we measured mRNA expression of several nAchR subunits and acetylcholine esterase (Ache) in gastrocnemius muscles of 4-week, 8-week and 1-year-old GDE5dC471 mice. Ache, nAchRα1 and nAchRε were up-regulated in GDE5dC471 mice relative to age-matched control mice (Fig 4A). As noted also, nAchRγ expression was equivalent between 4-week-old GDE5dC471 and control mice (Fig 4A). Next, we examined morphological alterations of nAchR localized at the myotube surface of the longitudinal section of gastrocnemius muscles in 1-year-old GDE5dC471 mice and in age-matched control mice using α-bungarotoxin staining. We found no differences in morphology between GDE5dC471 and control mice (Fig 4B).

### Primary alternation of gene expression in GDE5dC471 mice skeletal muscle

**Fig 5:**
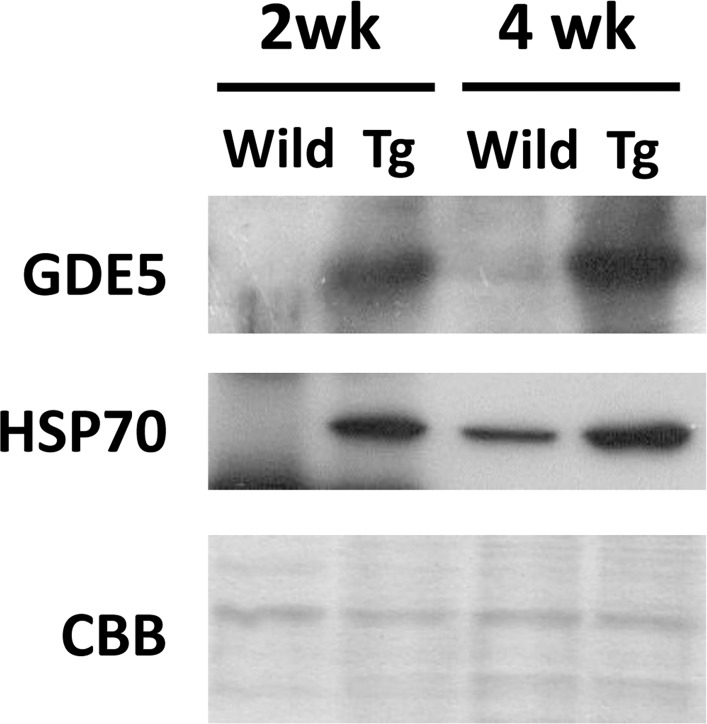
HSP70 protein expression in GDE5dC471 mice. Total protein from gastrocnemius muscle of 2-week-old and 4-week-old GDE5dC471 mice (Tg) and age-matched control mice (Wild) was subjected to SDS-PAGE followed by Western blotting using anti-GDE5 and anti-HSP70 antibodies. The filter was stained with Coomassie Brilliant Blue (CBB) as a control of protein loading.

**Fig 6:**
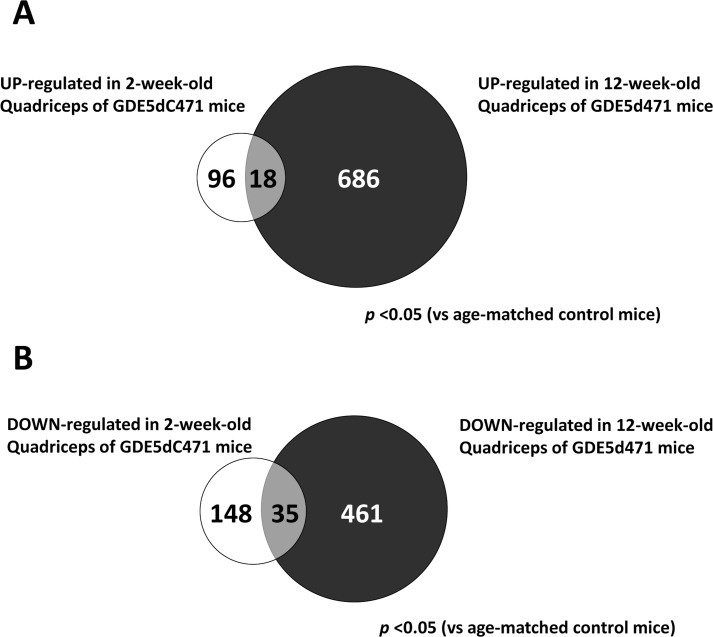
Two transcriptomes of quadriceps muscle in 2-week-old and 12-week-old of GDE5dC471 mice. The venn diagram shows genes that are altered in the quadriceps muscle of 2-week-old and 12-week-old GDE5dC471 mice. *A*, Of a total 96 genes up-regulated in quadriceps muscle of 2-week-old GDE5dC471 mice (*p*<0.05), the expression of 18 genes were also increased in that of 12-week-old. On the contrary (*B*), the expression of 35 genes were also down-regulated in quadriceps muscle of 12-week-old GDE5dC471 mice.

**Fig 7:**
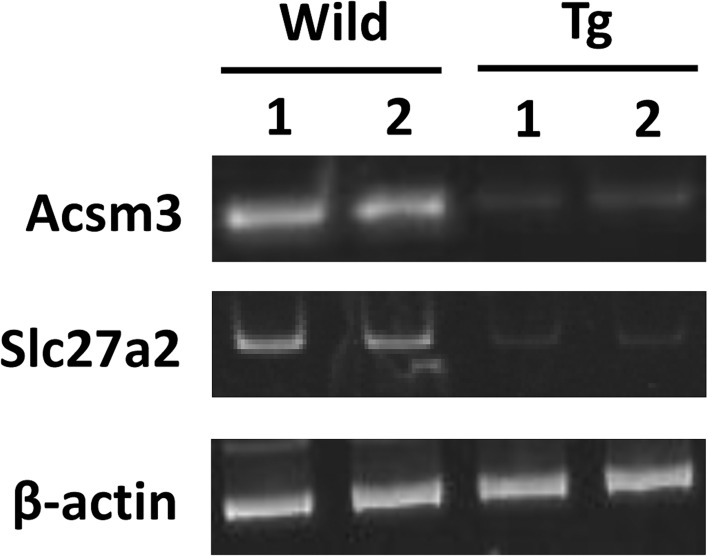
mRNA expression associated with lipid metabolism in GDE5dC471 mice. Semiquantitative RT-PCR was performed to determine mRNA levels of Acsm3 and Slc27a2 in quadriceps muscle of 2-week-old GDE5dC471 mice. The level of β-actin transcript was used for normalization.

In our previous study, mRNA expression analyses of the quadriceps of 12-week-old GDE5dC471 mice indicated that mRNAs for inflammatory cytokines and cellular stress (glutathione *s*-transferases) were up-regulated [30]. To further explore the mechanism of skeletal muscle atrophy development in GDE5dC471 mice, we examined 70 KD heat shock protein (HSP70) expression in type II fiber-rich skeletal muscle of 2-week-old and 4-week-old GDE5dC471 mice. HSP70 protein expression was markedly increased in 2-week-old GDE5dC471 mice compared with age-matched control mice (Fig 5), suggesting that this stress response plays an important role during early stages of skeletal muscle atrophy development due to GDE5dC471 overexpression. Thus, we expected that *in vivo* skeletal muscle factors that sensitively respond to GDE5dC471 expression can be identified in 2-week-old GDE5dC471 mice and performed DNA microarray analysis (whole-mouse-genome microarray) using RNA samples from type II fiber-rich skeletal muscle (quadriceps) of 2-week-old GDE5dC471 and control mice to isolate genes that are differentially expressed in response to GDE5dC471 overexpression. Ninety-six genes were up-regulated and 148 genes were down-regulated in 2-week-old GDE5dC471 mice relative to age-matched control mice (*p*<0.05) (Fig 6A and 6B). We then compared the transcriptomes of 2-week-old and 12-week-old GDE5dC471 mice to age-matched control mice and identified 18 genes and 35 genes that were significantly up-regulated and down-regulated, respectively, in both 2-week-old and 12-week-old GDE5dC471 mice (Fig 6A and 6B). Heat shock proteins (HSPs) including Hspa1a, Hspa8, Hspa4l, and Hsp110 were actually up-regulated along with other stress response markers, such as Fkbp5, Arrdc2, Gadd45g, Mt1 [34, 35], and Sesn2 (Table 3). Sesn2 is a genotoxic stress marker [36] and the gene product Sestrin 2 is considered to inhibit mTOR signaling that negatively regulates autophagy [37, 38], suggesting that increased Sesn2 expression facilitates clearance to protect against abnormal protein accumulation. Moreover, acyl-CoA synthetase (Acsm3) and a free-fatty acid transporter (Slc27a2) were down-regulated in 2-week-old (Table 3). We also compared mRNA expression of those in gastrocnemius muscles of 2-week-old GDE5dC471 mice with age-matched control mice. Acsm3 and Slc7a2 were down-regulated in GDE5dC471 mice relative to age-matched control mice, indicative of decreased fatty acid utilization [39, 40] in skeletal muscles of GDE5dC471 mice (Fig 7). Finally, we transfected GDE5dC471 into mouse myoblastic C2C12 myoblasts and examined HSP70 expression in these cells. HSP70 protein expression was actually increased in C2C12 myoblasts and myotubes overexpressing GDE5dC471 (S5 Fig), suggesting that GDE5dC471 is possibly involved in the cellular stress in the skeletal muscles *in vivo* in a direct manner during early stages of skeletal muscle development.

**Table 3:** Effect of GDE5dC471 over expression in type II fiber-rich skeletal muscles (quadriceps).

Gene ID	Gene symbol	Gene description	Fold change	P value
**Cell defence and stress response**				
NM_010220	Fkbp5	FK506 binding protein 5	2.26	0.000
NM_027560	Arrdc2	arrestin domain containing 2	1.96	0.000
NM_011817	Gadd45g	growth arrest and DNA-damage-inducible 45 gamma	1.74	0.000
NM_013559	Hsp110	heat shock protein 110	1.85	0.000
NM_010479	Hspa1a	heat shock protein 1A	2.47	0.000
NM_011020	Hspa4l	heat shock protein 4 like	1.63	0.001
NM_031165	Hspa8	heat shock protein 8	1.75	0.000
AK005714	Hspb9	hypothetical Heat shock hsp20 (alpha crystallin) proteins family containing protein	2.39	0.000
NM_019946	Mgst1	microsomal glutathione S-transferase 1	0.61	0.001
BC027262	Mt1	metallothionein 1	2.24	0.000
NM_144907	Sesn2	sestrin 2	5.83	0.000
**Energy metabolism**				
NM_009463	Ucp1	uncoupling protein 1 (mitochondrial, proton carrier)	0.16	0.000
**Glucose strage and metabolism**				
NM_177741	Ppp1r3b	protein phosphatase 1, regulatory (inhibitor) subunit 3B	0.45	0.000
NM_146118	Slc25a25	solute carrier family 25 (mitochondrial carrier, phosphate carrier), member 25	1.98	0.000
NM_145572	Gys2	glycogen synthase 2	0.57	0.000
**Lipid strage and metabolism**				
NM_007469	Apoc1	apolipoprotein C-I	0.29	0.000
NM_212441	Acsm3	acyl-CoA synthetase medium-chain family member 3	0.24	0.000
NM_008493	Lep	Leptin	0.32	0.000
NM_011146	Pparg	peroxisome proliferator activated receptor gamma	0.67	0.006
NM_022984	Retn	Resistin	0.59	0.000
NM_011978	Slc27a2	solute carrier family 27 (fatty acid transporter), member 2	0.18	0.000
NM_053200	Ces3	carboxylesterase 3	0.26	0.000
**Secreted proteins**				
NM_146125	Itpka	inositol 1,4,5-trisphosphate 3-kinase A	0.35	0.000
NM_023125	Kng1	kininogen 1	0.24	0.000
NM_031192	Ren1	renin 1 precursor	1.93	0.003
NM_170727	Scgb3a1	secretoglobin, family 3A, member 1	9.58	0.000
NM_198190	Ntf5	neurotrophin 5	1.40	0.020
**Structural proteins**				
NM_010230	Fmn1	formin 1	8.72	0.000
NM_009597	Accn2	amiloride-sensitive cation channel 2, neuronal	2.04	0.000
AF221104	Kifc5c	kinesin-related protein KIFC5C	25.76	0.000

DNA microarray analysis was repeated with the Cy3 and Cy5 dyes reversed (a dye swap), and fold change (Fold) represents the average of mRNA expression level in 2-week-old of GDE5dC471 transgenic mice relative to age-matched control mice.

## Discussion

In this study, transgenic mice overexpressing GDE5dC471 in skeletal muscles showed reduced type II-rich skeletal muscle mass and consistently smaller cross-sectional areas of type II-rich fibers from at least 8 weeks of age. In addition, decreases in passive motor function and increased adipose tissue weight were observed in 8-week-old and 1-year-old GDE5dC471 mice. On the other hand, type I-rich soleus muscle mass of GDE5dC471 transgenic mice was similar to that of age-matched control mice from juvenile to middle-aged. The type II fiber-specific atrophy that is characteristic of GDE5dC471 mice may be because endogenous GDE5 is highly expressed in type II-rich skeletal muscle [30]. Alternatively, since we used human skeletal muscle α-actin to specifically overexpress GDE5dC471 in skeletal muscle [30], the GDE5dC471 gene may be preferentially expressed in type II skeletal muscle.

Previous studies have indicated a close relationship between skeletal muscle atrophy and the degeneration of NMJs that are located at pre- and post-synapses [15–17]. In skeletal muscle, nAchRs are formed as pentameric subunits of (α1)2βδ (γ: fetal period) or (α1)2βδ (ε: mature period), and are located in post-synaptic membrane [21, 41–43]. mRNA expression of each subunit changes throughout development, for example, the nAchRε subunit was constantly expressed from youth to old age in the hindlimb muscles of FBNF1 rats, while the nAchRα and nAchRγ subunits were up-regulated with age [44]. On the other hand, all nAchR subunits were transiently up-regulated in the gastrocnemius muscles of surgically denervated rats, but mRNA levels returned to normal after spontaneous reinnervation [23], whereas mRNA expression of nAchRα1, γ, and ε subunits is increased in the diaphragm of Duchenne muscular dystrophy model mice in response to the destabilization of NMJs via congenital defects in dystrophin [33, 45]. Taken together, nAchR subunit up-regulation observed in skeletal atrophy models represents a compensatory response to decreased neuromuscular transmission by denervation of muscle or degeneration of NMJs.

In the present study, we found that nAchRα1, γ, and ε subunits were up-regulated in the gastrocnemius muscles of 8-week-old and 1-year-old GDE5dC471 mice, showing a similar feature as those in Duchenne muscular dystrophy model mice. However, NMJs were not morphological altered in GDE5dC471 mice even in 1-year-old mice, suggesting a distinct mechanism of up-regulation of genes related to NMJs from Duchenne muscular dystrophy model. On the other hands, various types of cellular stress (e.g., oxidative stress and chronic inflammation) are supposed to be primary causes of skeletal muscle atrophy [27, 28]. In our previous study, genes associated with inflammatory cytokines and cellular defenses, such as interleukin 1-β or glutathione *s*-transferases, were up-regulated in the quadriceps muscles of 12-week-old GDE5dC471 mice [30]. Hence, we examined the possibility that skeletal muscle atrophy in GDE5dC471 mice is mainly induced by cellular stress signaling. Expression of the molecular chaperone HSP70 is modulated by a variety of stress, such as heat shock, inflammation, or hypoxic state, and to intracellular accumulation of abnormal proteins [46, 47]. We found a dramatic increase in HSP70 protein expression in the gastrocnemius muscles of 2-week-old GDE5dC471 mice without a reduction in skeletal muscle mass. Moreover, C2C12 myoblasts and myotubes overexpressing GDE5dC471 showed high expression of HSP70 protein, suggesting that GDE5dC471 protein overexpressed in these cells is recognized as an abnormal protein. In fact, both HSP70 family genes (Hspa1a and Hspa8) and an HSP40 family member (Hapa4l) were up-regulated in the quadriceps muscles of 2-week-old GDE5dC471 mice possibly as a reaction against abnormal protein aggregation [48], because HSP40 reportedly functions as the co-chaperone for HSP70 and HSP110 (Hsp110), which are synergistically functional when both HSP40 and HSP70 promote large-scale protein disaggregation [49, 50]. On the other hands, the endoplasmic reticulum stress response also plays an important role as a defense against abnormal protein aggregation [50]. However, we found no change in the expression levels of endoplasmic reticulum stress response genes, including ATF6 and IRE1, and atrogin-1, MuRF1 and Cbl-b those are involved in the ubiquitin proteasome pathway that degrades abnormal proteins [51].

Although NMJs were not morphological altered in GDE5dC471 mice in 1-year-old mice, the numbers of centrally located nuclei and apoptotic cells were increased in the type II fibers of 8-week-old and 1-year-old GDE5dC471 mice. Importantly, increased mRNA expression of nAchR subunits was observed even in 4-week-old GDE5dC471 mice that did not show altered skeletal muscle mass. Taken together, these observations suggest that the increased nAchR subunits expression in the skeletal muscles of GDE5dC471 mice may indicate an adaptation against cellular stress signaling and/or skeletal muscle fiber degeneration and regeneration. Thus, GDE5dC471 mice can be applied for basic research on certain human myopathies that exhibit compensatory expression of NMJs components that are a possible consequence of increased cellular stress rather than NMJ degradation. The current study characterized the pathophysiological features of GDE5dC471 mice with type II skeletal muscle atrophy without a neurotransmission disorder and further suggests the possibility that intracellular abnormal protein accumulation is associated with primary skeletal muscle pathology.

## Supporting Information

S1 File Supporting Materials and Methods. (DOCX)

S1 Fig Time-course increase in centrally localized myonuclei in type II fiber-rich muscles of GDE5dC471 mice. Mean ratio of centrally localized myonuclei per myofiber number in transverse sections of hematoxylin and eosin (H&E) stained gastrocnemius muscle of 4 and 8-week-old and 1-year-old GDE5dC471 mice (Tg) and age-matched control mice (Wild). 4 and 8-week-old: n = 4, and 1-year-old: n = 6. Data represent mean ± SD. *p<0.05, **p<0.01.
(PDF)

S2 Fig mRNA expression of mitochondria- and glycolysis-related genes in gastrocnemius muscle of 1-year-old of GDE5dC471 mice. Total RNA from gastrocnemius muscle of 1-year-old of GDE5dC471 mice (Tg) and age-matched control mice (Wild) was subjected to quantitative PCR to examine mRNA expression level of genes related to mitochondrial functions (A) or glucose metabolism (B). n = 5, Data represent mean ± SE. **p<0.01.
(PDF)

S3 Fig mRNA expression of myosin heavy chain in gastrocnemius muscle of 1-year-old of GDE5dC471 mice. Quantitative PCR was performed to compare myosin heavy chain (MHC) mRNA expressions in gastrocnemius muscle of 1-week-old of GDE5dC471 mice (Tg) with age-matched control mice (Wild). n = 5, Data represent mean ± SE.
(PDF)

S4 Fig Evaluation of spontaneous locomotive activity in 1-year-old of GDE5dC471 mice. Spontaneous locomotive activity was examined by running wheel test. Mice were transferred to cages with a running wheel and monitored for the number of wheel revolutions made for 3 days (n = 3). There was no statistical significance between GDE5dC471 mice (Tg) and age-matched control at 1-week-old. Data represent mean ± SE.
(PDF)

S5 Fig GDE5dC471 overexpression in C2C12 myoblasts and myotubes. A, C2C12 cells were transiently transfected with a construct for GDE5dC471. After transfection, the C2C12 myoblasts and myotubes were fixed and stained with anti-GDE5 and anti-HSP70 antibodies. Primary antibody was visualized by fluorescein isothiocyanate- or Cy3-conjugated secondary antibody. *Green*; GDE5, *red*; HSP70 and *blue*; DAPI.
(PDF)

## Abbreviations

GDE5: glycerophosphodiesterase 5
NMJ: neuromuscular junction
nAchR: nicotinic acetylcholine receptor
TUNEL: TdT-mediated dUTP nick end labeling
HSP: heat shock protein
Ache: acetylcholine esterase
PCR: polymerase chain reaction
