# Supplementary material for: Time Course Analysis of Skeletal Muscle Pathology of GDE5 Transgenic Mouse
Source: PLoS One. 2016 Sep 22;11(9):e0163299. doi: 10.1371/journal.pone.0163299 (PMC5033411; doi:10.1371/journal.pone.0163299)
Supplement: S1 File — (DOCX) [file pone.0163299.s001.docx]

# Supporting Materials and Methods

Centrally localized myonuclei ratio

Gastrocnemius muscle of GDE5dC471 mice and age-matched control mice at 4 and 8-week, and 1-year old of age were fixed in neutral buffered 10% formalin, and processed by staining paraffin-embedded transverse sections (2 μm) with hematoxylin and eosin (H&E). In order to calculate the ratio of centrally localized myonuclei to muscle fiber number, counting more than 300 myofibres per mouse were performed using the H&E staining section.

Cell cultures, transfection and immunofluorescence microscopy

Mouse myoblastic C2C12 cells were cultured in DMEM supplemented with 10% FCS, 100 units/ml penicillin, and 100 μg/ml streptomycin under a humidified atmosphere of 5% CO2 in air at 37 °C. A cDNA encoding GDE5ΔC471 (amino acids 1–470) was subcloned into the mammalian expression vector pEF/myc/cyto (Invitrogen), generating pEF-GDE5ΔC471. DNA transfections were performed using GeneJuice Transfection Reagent (Millipore, Bedford, MA) according to the manufacturer's instructions. After the C2C12 cells reached confluence, the medium was replaced with myogenic medium, consisting of DMEM supplemented with 2% horse serum, and these cells were further cultured for 2 days. C2C12 myoblasts and myotubes were fixed with 4% paraformaldehyde and 4% sucrose in 0.2 m sodium phosphate buffer (pH 7.2) for 30 min and blocked with 1.5% normal goat serum in PBS for 1 h at room temperature, followed by incubation with rabbit anti-GDE5 antibody (Okazaki, et al) and anti-HSP70 mouse monoclonal antibody (Santa Cruz Biotechnology, Santa Cruz, CA), which was visualized with fluorescein isothiocyanate- or Cy3-conjugated secondary antibodies (GE Healthcare). DAPI was used for nuclear staining. Epifluorescent images were captured with a CCD camera (Hamamatsu) mounted on a Nikon Eclipse E600 microscope.

Quantitative PCR analyses

Realtime PCR analyses were performed on total RNA, which was extracted from dissected gastrocnemius muscles of 1-year-old GDE5dC471 and age-matched control mice, prepared with RNeasy Lipid Tissue Mini Kit (Qiagen, Hilden, Germany) according to the instructions of the manufacturer. The reverse transcriptase reaction was carried out with 1 μg of total RNA as a template to synthesize cDNA using RevaTra Ace (Toyobo, Osaka, Japan) reverse transcriptase. To quantify PCR products, cDNA and specific primers were added to THUNDERBIRD™SYBR ®qPCR Mix (Toyobo) to give a total reaction volume of 15 μL, and PCR reactions were performed using StepOnePlus^TM^ (Applied Biosystems, Foster City, CA). The PCR reactions were initiated with denaturation for 10 min at 95°C, followed by amplification with 40 cycles each for 15 s at 95 °C, and extended for 1 min at 60 °C. The primers used for realtime PCR analysis were as follows: MHC-I, sense, 5’-CCAAGGGCCTGAATGAGGAG-3’, and antisense, 5’-GCAAAGGCTCCAGGTCTGAG-3’; MHC-IIa, sense, 5’-AAGCGAAGAGTAAGGCTGTC-3’, and antisense, 5’-GTGATTGCTTGCAAAGGAAC-3’; MHC-IIb, sense, 5′-ACAAGCTGCGGGTGAAGAGC-3′, and antisense, 5′-CAGGACAGTGACAAAGAACG-3′; MHC-IIx, sense,5′-CCAAGTGCAGGAAAGTGACC-3′, and antisense, 5′-AGGAAGAGACTGACGAGCTC-3′; αGPD, sense, 5′-CAACTTTCGCATCACTGTGG-3′, and antisense, 5′-AGCTGCTCAATGGACTTTCC-3′; Cs, sense, 5′-AGCTCATGCGTTTGTACCTC-3′, and antisense, 5′-CTCATCTGACACGTCTTTGC-3′; PGC-1α, sense, 5′-ATGTGTCGCCTTCTTGCTC-3′, and antisense, 5′-TGTCTGTAGTGGCTTGATTC-3′; L19, sense, 5′-GGCATAGGGAAGAGGAAGG-3′, and antisense, 5′-GGATGTGCTCCATGAGGATGC-3′.

Samples were normalized according to L19 mRNA levels.

Running wheel test

Spontaneous locomotive activity in 1-year-old GDE5dC471 and control mice was assessed by RW-15 running wheel apparatus (MELQUEST, Toyama, Japan). Mice were transferred to individual cages with the apparatus and corrected output signal of 1 pulse per revolution by an on/off signal of microswitch for 3 days, and calculated the mean revolution number per day.

Statistical analysis

To determine significant differences in pathophysiology, parametric comparisons between GDE5dC471 mice and age-matched control mice were carried out the two-tail unpaired t-test. Timecourse data was analyzed with a two-way ANOVA followed by the two-tail unpaired t-test for evaluation the statistical significance between GDE5dC471 mice and age-matched control mice at 4 and 8-week, and 1-year old of age. Statistical analysis was executed with Prism 7 version 7.01 software (GraphPad Software, Inc.). *p* values of < 0.05 (two sided) were considered statistically significant.
