# Supplementary figures and images for: Time Course Analysis of Skeletal Muscle Pathology of GDE5 Transgenic Mouse
Source: PLoS One. 2016 Sep 22;11(9):e0163299. doi: 10.1371/journal.pone.0163299 (PMC5033411; doi:10.1371/journal.pone.0163299)

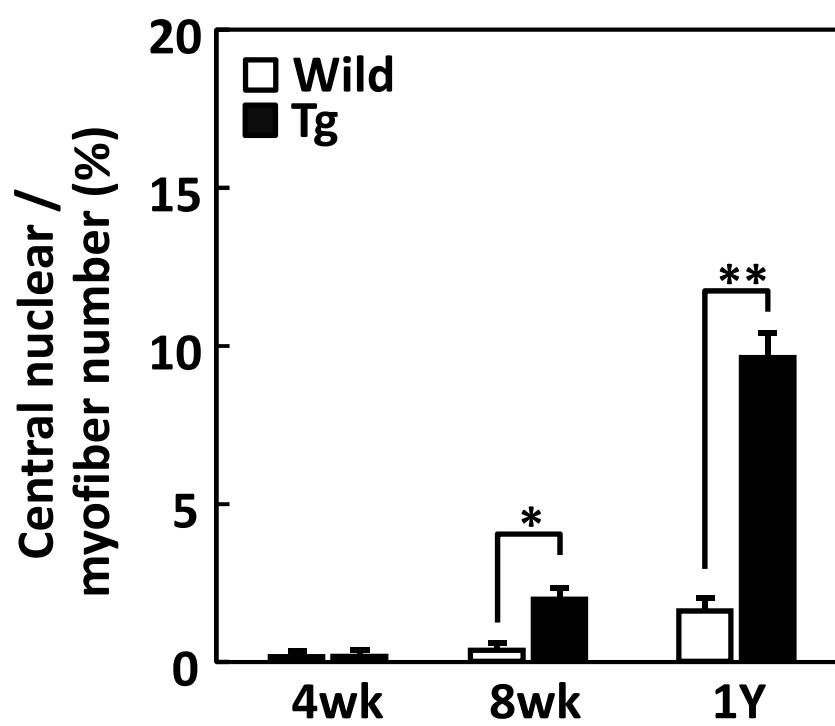

Supplement: S1 Fig — Mean ratio of centrally localized myonuclei per myofiber number in transverse sections of hematoxylin and eosin (H&E) stained gastrocnemius muscle of 4 and 8-week-old and 1-year-old GDE5dC471 mice (Tg) and age-matched control mice (Wild). 4 and 8-week-old: n = 4, and 1-year-old: n = 6. Data represent mean ± SD. *p<0.05, **p<0.01. (PDF) [file pone.0163299.s002.pdf]

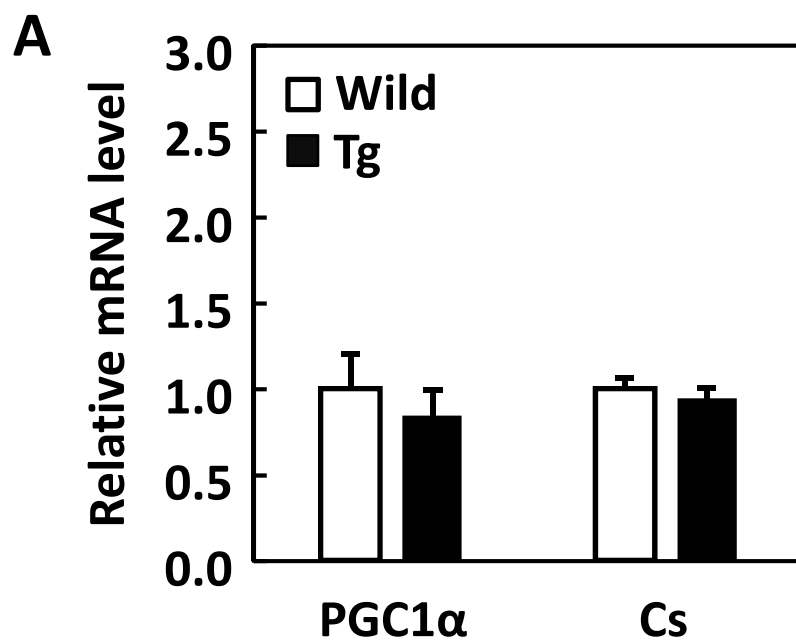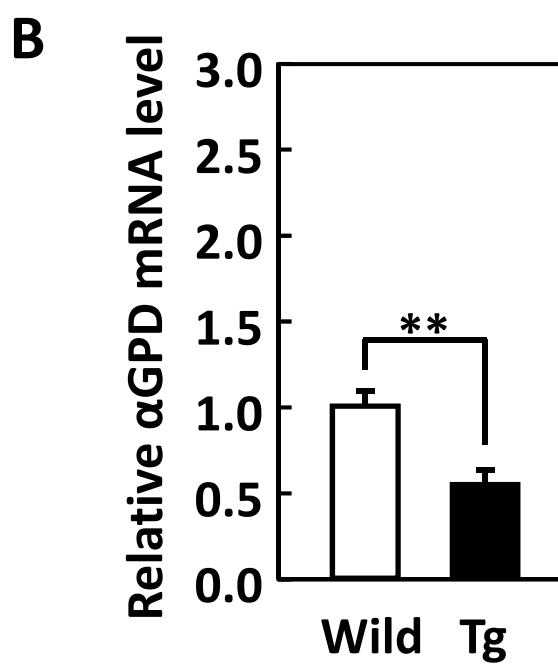

Supplement: S2 Fig — Total RNA from gastrocnemius muscle of 1-year-old of GDE5dC471 mice (Tg) and age-matched control mice (Wild) was subjected to quantitative PCR to examine mRNA expression level of genes related to mitochondrial functions (A) or glucose metabolism (B). n = 5, Data represent mean ± SE. **p<0.01. (PDF) [file pone.0163299.s003.pdf]

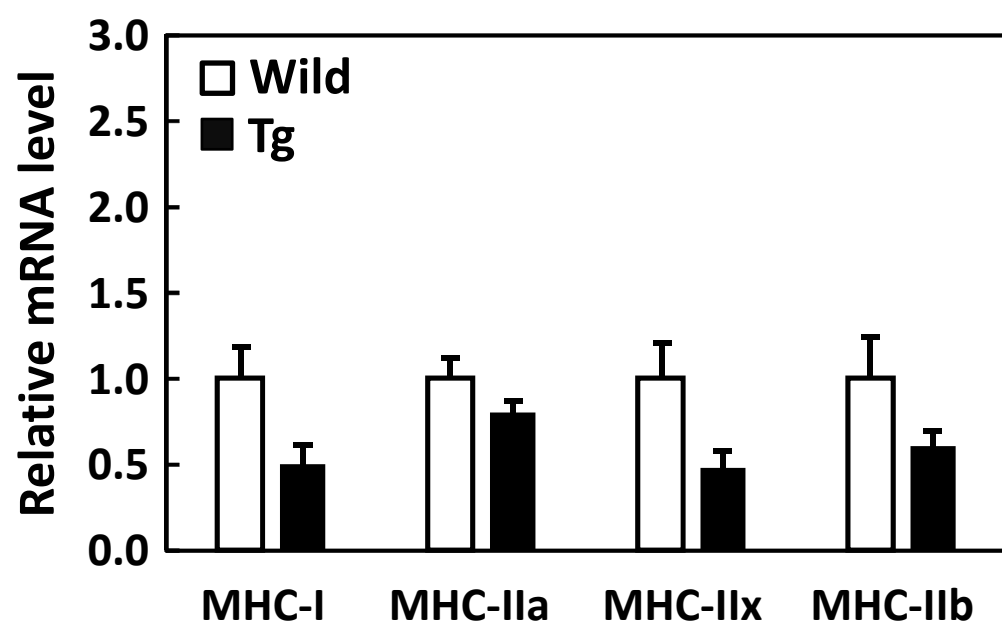

Supplement: S3 Fig — Quantitative PCR was performed to compare myosin heavy chain (MHC) mRNA expressions in gastrocnemius muscle of 1-week-old of GDE5dC471 mice (Tg) with age-matched control mice (Wild). n = 5, Data represent mean ± SE. (PDF) [file pone.0163299.s004.pdf]

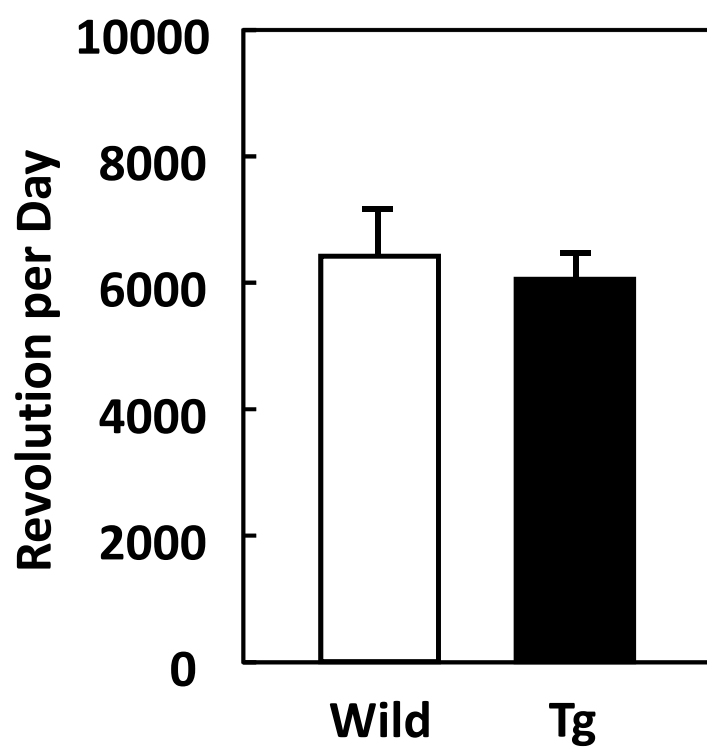

Supplement: S4 Fig — Spontaneous locomotive activity was examined by running wheel test. Mice were transferred to cages with a running wheel and monitored for the number of wheel revolutions made for 3 days (n = 3). There was no statistical significance between GDE5dC471 mice (Tg) and age-matched control at 1-week-old. Data represent mean ± SE. (PDF) [file pone.0163299.s005.pdf]

**A**

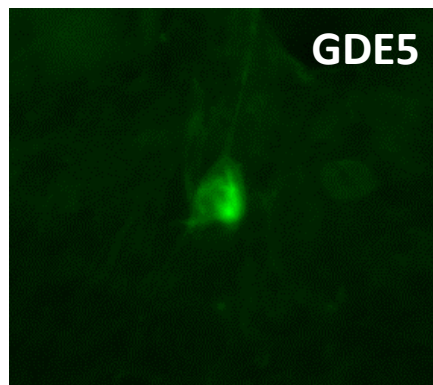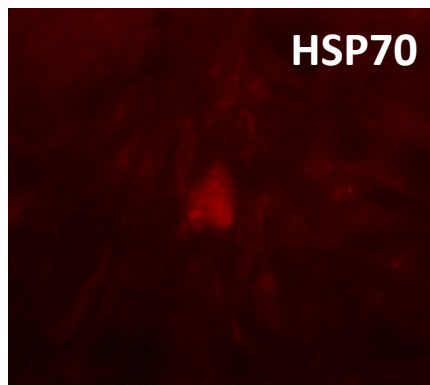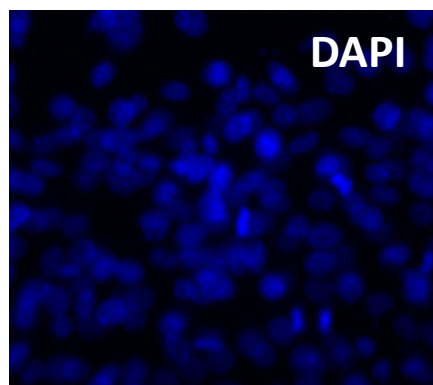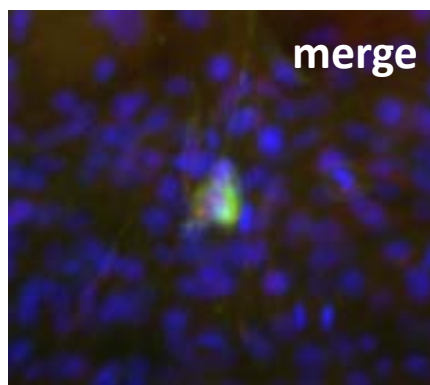

**B**

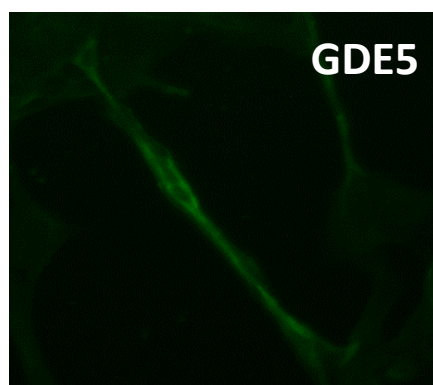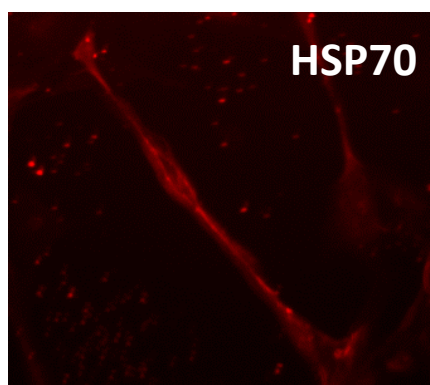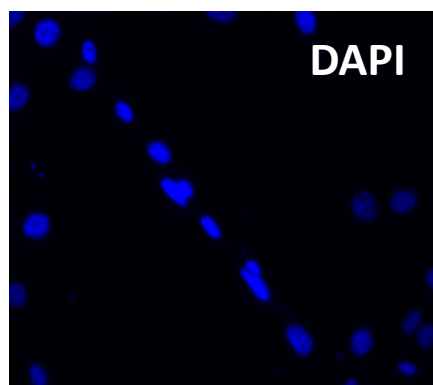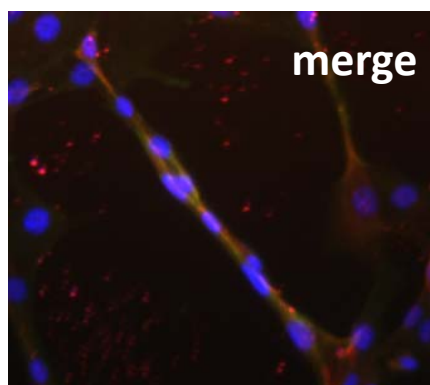

Supplement: S5 Fig — A, C2C12 cells were transiently transfected with a construct for GDE5dC471. After transfection, the C2C12 myoblasts and myotubes were fixed and stained with anti-GDE5 and anti-HSP70 antibodies. Primary antibody was visualized by fluorescein isothiocyanate- or Cy3-conjugated secondary antibody. Green; GDE5, red; HSP70 and blue; DAPI. (PDF) [file pone.0163299.s006.pdf]
